# Update on the biology and ecology of *Culicoides* species in the South-West region of Cameroon with implications on the transmission of *Mansonella perstans*

**DOI:** 10.1186/s13071-019-3432-9

**Published:** 2019-04-11

**Authors:** Samuel Wanji, Dizzle Bita Tayong, Rene Ebai, Vera Opoku, Chi Anizette Kien, Winston Patrick Chounna Ndongmo, Abdel Jelil Njouendou, Raymond Nsaidzedze Ghani, Manuel Ritter, Yaw Alex Debrah, Laura E. Layland, Peter A. Enyong, Achim Hoerauf

**Affiliations:** 1 Parasite and Vector Biology Research Unit (PAVBRU), Department of Microbiology and Parasitology, University of Buea, P.O. Box 63, Buea, Cameroon; 2 Research Foundation for Tropical Diseases and the Environment (REFOTDE), P.O. Box 474, Buea, Cameroon; 3 Kumasi Centre for Collaborative Research (KCCR), Kumasi, Ghana; 4 Institute of Medical Microbiology, Immunology and Parasitology (IMMIP), University Hospital Bonn, Bonn, Germany; 5 German Centre for Infection Research (DZIF), Partner Site, Bonn-Cologne, Bonn, Germany

**Keywords:** *Culicoides* species, Relative abundance, Breeding sites, Biting preferences, *Culicoides milnei*, *Mansonella perstans*

## Abstract

**Background:**

*Culicoides* (Diptera; Ceratoponidae) are tiny, stout, blood-sucking flies with a near worldwide distribution. When present, they are often considered a biting nuisance but in addition, they are involved in the transmission of pathogens to humans, domestic and wild animals. Data on *Culicoides* species in the South-West region of Cameroon dates back to the 1950s. Over the decades, ecological transformation due to agriculture and deforestation may have affected the population dynamics of *Culicoides* and therefore our study provides an update of their bio-ecology in the region. Furthermore, the role of various *Culicoides* species in the transmission of parasitic filariae of the genus *Mansonella* remains inconclusive in this region. This study was designed to address these unknown issues and expand on current scientific knowledge.

**Results:**

Eight species of *Culicoides* (*C. bedfordi*, *C. inornatipennis*, *C. fulvithorax*, *C. grahamii*, *C. imicola*, *C. milnei*, *C. neavei* and *C. kumbaensis*) were collected using light traps and human baits. *Culicoides grahamii* was the most abundant species, followed closely by *C. milnei*. Three species (*C. milnei*, *C. grahamii* and *C. inornatipennis*) were common in all observed larval development sites. Only four species (*C. inornatipennis*, *C. fulvithorax*, *C. grahamii* and *C. milnei*) were collected on humans. Anthropophilic species were more abundant (*P* < 0.001) in the evening (4–7 pm) when compared to the morning collections (6–9 am). After overnight fly collections using a drop trap with a human microfilaremic donor, *C. milnei* emerged as the potential host for transmitting *Mansonella perstans*. Substantial heterogeneity was observed between the trap visiting cycles of the various species (*P *< 0.001). The biting cycle of the main vector, *C. milnei*, showed two peaks (10–11 pm and 4–5 am), the highest being 10–11 pm.

**Conclusions:**

The *Culicoides* fauna of the South-West region of Cameroon has not changed significantly since the 1950s. *Culicoides milnei* was demonstrated to be the major vector of *M. perstans* in this part of Cameroon. It is essentially a nocturnal species which peaks in abundance between 10 and 11 pm.

**Electronic supplementary material:**

The online version of this article (10.1186/s13071-019-3432-9) contains supplementary material, which is available to authorized users.

## Background

Biting midges belong to the family Ceratopogonidae with the most common species being *Culicoides* [1]. Over 1400 species have been described in the genus *Culicoides* and, with the exception of Antarctica and New Zealand, they are prevalent worldwide [2, 3]. The distribution, abundance and seasonal occurrence of these holometabolous flies is determined by the availability of moisture-rich habitats that are essential for the development of immature stages [2]. They are associated with aquatic or semiaquatic habitats, e.g. mud or moist soil around streams, ponds and marshes [1]. They are also associated with the dung of various animals such as cattle, buffalo and equines, and breed in rotting fruit, cacti, banana stems and leaf detritus [1]. The presence of animals, especially livestock such as cattle, horses and sheep also play an important role in the abundance/distribution of *Culicoides*. Biting midges inflict painful bites and suck the blood of their hosts, which include humans, livestock and wild animals [3].

In areas where *Culicoides* are abundant they may constitute a biting nuisance to humans, domestic and wild animals [2, 4–7]. Their bites are sometimes associated with allergic skin reactions which in some individuals may result in urticaria. *Culicoides* are diverse in terms of both affected species and the pathogens they transmit. They are involved in the transmission of animal and human viruses as well as animal and human filariae [8].

The first definite association between flies of the family Ceratopogonidae and the transmission of filarial infections in Cameroon was made by Sharp in 1927 [9, 10] who reported on the development of *Acanthocheilonema perstans* (Manson, 1891) in *Culicoides grahamii* (Austen, 1909) and *Culicoides austeni* (Carter, Ingram & Macfie, 1920) in rainforest areas. In 1949, Henrard & Peel [11] and Chardome & Peel [12] suggested that Sharp was actually working with *Dipetalonema streptocerca* (Macfie & Corson, 1922) instead of *A. perstans*, now *Mansonella perstans*. Hopkins & Nicholas [13] also suggested that *C. grahamii*, *C. austeni* and *C. inornatipennis* (Carter, Ingram & Macfie, 1920) were potential vectors of *M. perstans* in South-West Cameroon and it remains unclear how abundant these species are in forest areas. Previously suitable ecological conditions for the *Culicoides* may have been modified due to human activity (land occupation for housing, farming, timber exploitation). Moreover, since these initial observations nearly half a century ago, few studies have updated either vector biology or the significance of the flies in disease transmission. *Mansonella perstans* has been reported to be endemic in Cameroon, with its distribution highly influenced by bioecology [14]. In the South-West region, where the prevalence of the parasite has not changed even after a decade of ivermectin mass drug administration (MDA), there is a need to investigate the vectors implicated in the transmission of the causative agent of the disease. Vector identification is an important step in the epidemiology of vector-borne diseases and information on the major vector species may provide a clearer indication of the disease distribution (or its potential distribution) and identify high-risk areas for human-vector contact. Of the 13 species of *Culicoides* previously identified in Cameroon, at least five feed on humans in South-West Cameroon [8, 15, 16]. Given the limited information on *Culicoides* species in South-West Cameroon and their possible role in the transmission of filarial parasites, this study was designed to determine species diversity of *Culicoides*, identify anthropophagous species, and elucidate their role in the transmission of *M. perstans*, an endemic and widespread filarial species in Africa.

## Methods

### Study design and study site

**Fig. 1:**
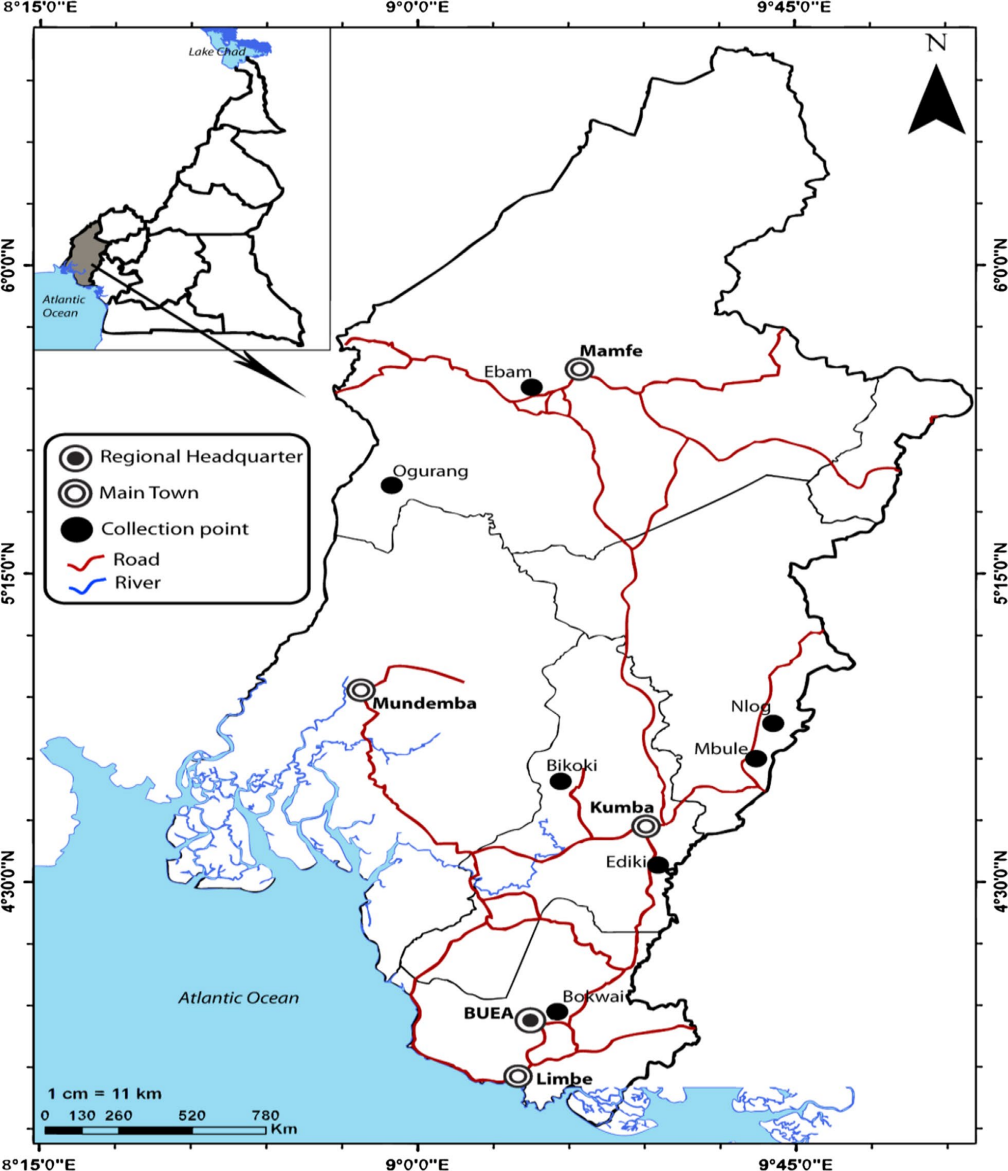
Map of the South-West region of Cameroon showing the study sites with the *Culicoides* collection points

A cross-sectional survey was carried out in seven selected communities of five health districts in the South-West region of Cameroon (Fig. 1) during the rainy season (May to September). These communities were Bikoki, Bokwai, Ebam, Ediki, Mbule, Nlog and Ogurang (Table 1). The study sites were characterized by the absence of industrial livestock, but individual families reared chickens, pigs and cattle (max. three animals/family). Temperatures during this season are around 20 °C with a relative humidity of 75–80% and daily precipitation of 6–14 mm/day [17]. Due to the volcanic soil of this region, the inhabitants farm plantains around their dwelling/communities. After the harvest, decaying plantains become larval development sites of *Culicoides* species.

**Table 1:** Grid-references of the study sites

Site	Longitude	Latitude	Altitude (m)
Bikoki	9.2851	4.7459	536
Bokwai	4.758703	4.171846	743
Ebam	9.2263233	5.7003367	611
Ediki	4.954336	4.543133	111
Mbule	5.153599	4.804900	741
Nlog	5.183033	4.875906	735
Ogurang	8.9600	5.4789	822

### Collection of adult *Culicoides* species using CDC miniature UV-light traps

Due to the difficult access to some study sites during the rainy season, there were variations in the number of overnight collections per site. Collections of midges were carried out using CDC miniature black UV-light traps (Model 512, John W. Hock Company, Gainesville, USA). The number of collections ranged from 2 nights in Nlog to 6 nights in Ediki. To determine species diversity, we collected adults and potential larval developmental material from which larvae were isolated and reared to adults. Four UV-light traps were set at strategic positions around human dwellings at each of the seven sites. Hourly collections were made from 6 pm to 6 am each working day. For all study sites, the content of each trap was emptied hourly into labelled plastic bottles which were stored in a cooler box and transported to the laboratory for *Culicoides* identification.

### Collection of adult *Culicoides* species using the human landing catch (HLC) technique

To determine which *Culicoides* species targeted humans, adult flies were collected using the HLC technique from members of four communities, Bokwai, Ediki, Mbule and Nlog (Fig. 1). HLC was carried out in the morning (6–9 am) and evening (4–7 pm) [15, 16]. This was done by four well trained collectors dressed in protective clothing against midges. The collectors were positioned in four different houses in each study site for 4 days of collection in each study month. Each collector was supplied with a torch to enable him to continue fly collection in darkness (6–7 pm). At collection sites the same collection effort was implemented: 3 h/morning and 3 h/evening for 4 days, making a total of 12 h collection for the morning periods and 12 h for the evening periods. *Culicoides* females seeking a blood meal were aspirated as soon as they landed on collectors. The aspirated midges were transferred immediately into well-labeled hourly-netted plastic cups and transported to the laboratory for morphological identification and preservation.

### Collections of larval developmental material

Potential breeding material, banana/plantain stems in the 1st and 2nd stage of decay, were collected from four communities (Bokwai, Ediki, Mbule and Nlog). Breeding material was graded as described by Hopkins [16]. In the 1st stage of decay, the stems are either upright or prostrate and have concentric rings of fibrous tissue separated by the softer decaying intermediary cells. The 2nd stage of decay is characterized by a remaining basal disk which is hard outside but contains a mass of rotting cells inside which has fallen down. The material was transported to the laboratory where the larvae were isolated and reared to adult stages [15]. In the laboratory, five stems of 30 cm in length and 20 cm in diameter were selected per site. *Culicoides* larvae were isolated from the compost material as previously described by Hopkins and colleagues [13, 15]. Briefly, *Culicoides* larvae were isolated at room temperature by washing the compost material using sieves of different pore sizes followed by magnesium sulphate flotation. The larvae were reared in the laboratory using a 12 h day/night lighting regime at 22 °C and 80% relative humidity on compost which had been heat sterilized at 70–90 °C for 20 min. Prior to collection of adult midges (emergence), the compost was placed in cages designed for that purpose: square shaped 900 cm^2^ boxes covered with very fine netting of pore size 0.5 mm.

### Collections of engorged *Culicoides* from a *M. perstans* microfilaremic volunteer using a drop trap

**Fig. 2:**
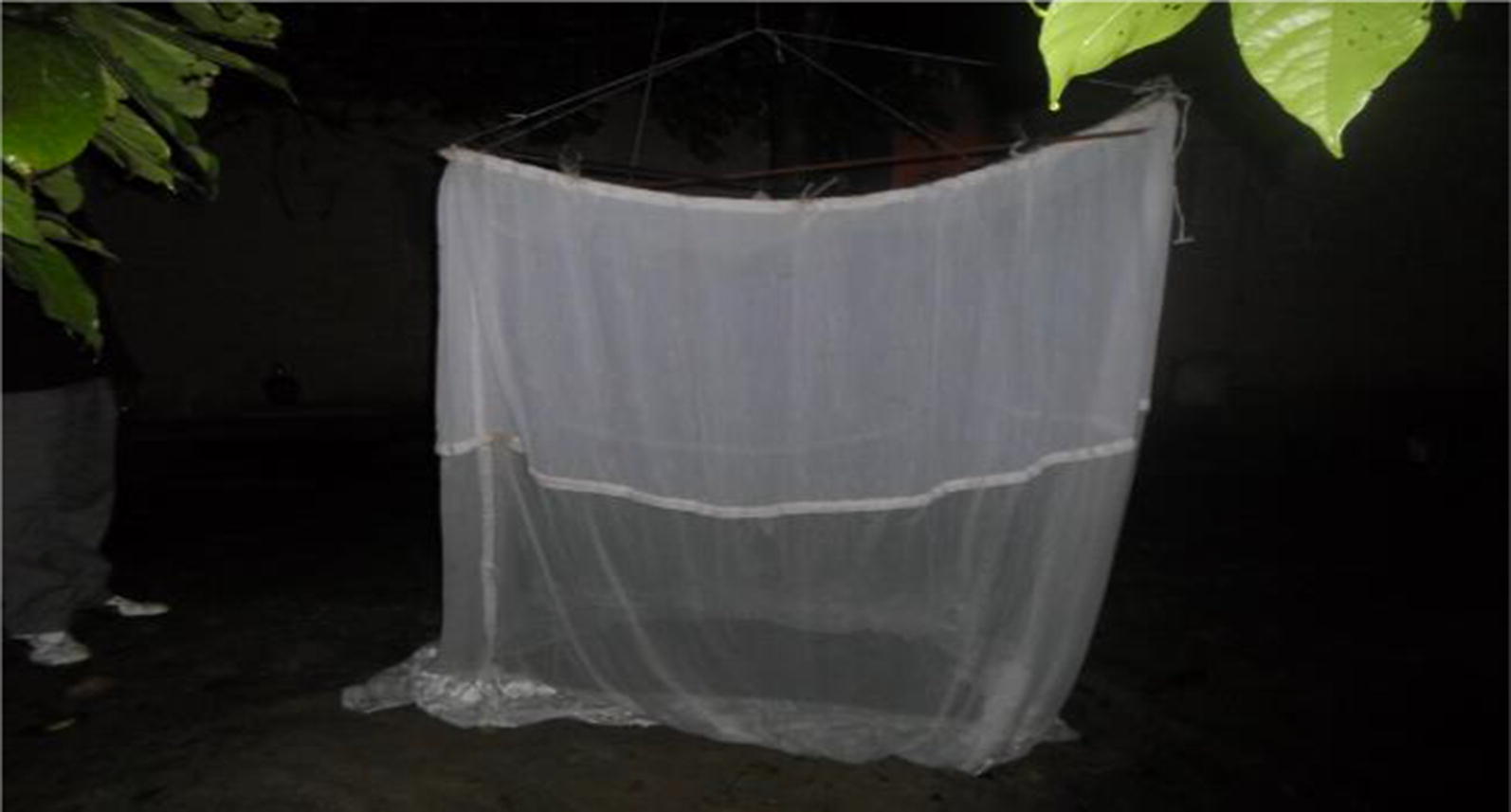
Rectangular drop trap set in the night in the human dwelling

To elucidate the role of major anthropophagic species in the transmission of *M. perstans*, overnight HLC using a drop trap was conducted at Ediki. Collections were done from 6 pm to 6 am for four nights. An *M. perstans* microfilaraemic volunteer sat under a rectangular netting cage trap (2 × 2 × 2 m; Fig. 2). The *M. perstans* status of the volunteer had been ascertained using the thick blood film technique [18]. The cage was raised for 10–15 min to allow contact between the host and midges and then lowered to trap the attracted midges. After about 12 min, the expected time for trapped midges to be fully blood-fed, engorged flies were gently aspirated and blown into well-labeled 50 ml falcon tubes 3/4 filled with plaster of Paris (POP). The POP formed an absorbent layer at the bottom of the tubes to retain moisture.

The midges were maintained in the tubes in the field using the protocol of Hopkins [15]. The collected midges were fed with 15% sugar solution and transported to the laboratory in a cooler box for maintenance. The *Culicoides* were maintained at 23 ± 2 °C and relative humidity (RH) of 75 ± 5%. A hole (0.5 cm in diameter) was made in the lid of each rearing tube to permit ventilation and feeding of the flies in captivity. This hole was covered with fine netting (pore size 0.5 mm) on which the sugar solution-moistened cotton was placed for fly-feeding. Flies were fed daily with 15% sugar solution soaked in cotton gauze. Additionally, 3–4 drops of distilled water were added daily using a 10 ml syringe to keep the tubes moist. Mortality was recorded daily, and the insects were identified and dissected 12 days post-feeding to retrieve infective larvae of *M. perstans*. During dissection, the head, thorax and abdomen were separated and gently teased open in individual dissection wells containing incomplete culture medium (RPMI-1640 medium; Sigma-Aldrich, Munich, Germany) supplemented with a 2% antibiotic cocktail (penicillin-streptomycin-neomycin; Thermo Fisher Scientific, Schwerte, Germany). The species that yielded infective larvae (L3) as well as any other developmental stages (L1 or L2) were noted and the numbers of larvae recorded.

### Morphological identification of adult *Culicoides* species

Morphological identification of *Culicoides* species was done by examination of the wing pigmentation pattern under a dissecting microscope. In cases difficulties were identifying species based on wing pattern proved inconclusive, other morphological characteristics such as maxillary palps inter-ocular space and male genitalia were used. A combination of identification keys were used for species identification [8, 19, 20].

### Data analysis

Data from fly collections, site of collections, identification and dissection were entered in a template designed in Epi info v.3.5.3 (CDC, Atlanta, USA) and exported to SPSS v.20 (IBM, Armonk, NY, USA) for statistical analysis. *Culicoides* diversity and abundance was expressed as the number of different species of *Culicoides* collected per site per force of work made at that site. The number of flies captured by a trap in a day (fly/trap/day) was expressed as the number of flies collected divided by the number of traps used divided by the number of collection days. The number of flies per person per day (fly/person/day) was expressed as the number of flies attempting to bite a collector divided by the product of the number of collection days and the number of collectors. The Chi-square test was used to compare the abundance of the different species across the communities as well as the proportions of adult species collected by the different trapping methods. *P*-values less than 0.05 were considered statistically significant.

## Results

### Species of *Culicoides* collected with CDC miniature UV-light traps

Eight *Culicoides* species were collected over a 4 month period at different time points at 7 sites: *C. bedfordi* (Ingram & Macfie, 1923), *C. inornatipennis*, *C. fulvithorax* (Austen, 1909), *C. grahamii*, *C. imicola* (Kieffer, 1913), *C. milnei* (Austen, 1909), *C. neavei* (Austen, 1912) and *C. kumbaensis* (Callot, Kremer, Mouchet & Bach, 1965) (Table 2). Generally, the most abundant species as determined with UV-light trapping was *C. grahamii* (*n* = 2865), with an abundance of 31.1 flies/trap/night. The species with the lowest fly/trap/night ratio was *C. imicola* with 0.01 fly/trap/night*. Culicoides grahamii* represented 41.6% of the 6889 specimens collected by this method and was closely followed by *C. milnei* (30.7%). The Chi-square test demonstrated a significant difference in the total number of flies collected at the different sites (*χ*^2^ = 7504.2, *df *= 42, *P* < 0.0001).

**Table 2:** *Culicoides* species collected using UV-light traps during May-September 2016 at 7 study sites in the South-West region of Cameroon

Species	Sites							Total (%)	Fly/trap/ night
	Bikoki (*n* = 12)	Bokwai (*n* = 12)	Ebam (*n* = 12)	Ediki (*n* = 24)	Mbule (*n* = 12)	Nlog (*n* = 8)	Ogurang (*n* = 12)	Night-traps (*n* = 92)	
*C. grahamii*	630 (52.5)	225 (18.8)	26 (2.2)	1249 (52.0)	578 (48.2)	3 (0.4)	154 (12.8)	2865 (41.6)	31.1
*C. milnei*	164 (13.7)	16 (1.3)	350 (29.2)	1025 (42.7)	519 (43.3)	6 (0.8)	38 (3.2)	2118 (30.7)	23.0
*C. fulvithorax*	14 (1.2)	0 (0)	82 (6.8)	35 (1.5)	42 (3.5)	1 (0.1)	981 (81.8)	1155 (16.8)	12.6
*C. inornatipennis*	12 (1)	273 (22.8)	14 (1.2)	63 (2.6)	80 (6.7)	9 (1.1)	35 (2.9)	486 (7.1)	5.3
*C. neavei*	14 (1.2)	0 (0)	49 (4.1)	0 (0)	9 (0.8)	0 (0)	47 (3.9)	119 (1.7)	1.3
*C. kumbaensis*	1 (0.1)	0 (0)	1 (0.1)	2 (0.1)	97 (8.1)	0 (0)	3 (0.3)	104 (1.5)	1.1
*C. bedfordi*	22 (1.8)	0 (0)	10 (0.8)	0 (0)	0 (0)	0 (0)	9 (0.8)	41 (0.6)	0.4
*C. imicola*	0 (0)	1 (0.1)	0 (0)	0 (0)	0 (0)	0 (0)	0 (0)	1 (0.01)	0
Total	857	515	532	2374	1325	19	1267	6889 (100)	74.9
Fly/trap/night	71.4	42.9	44.3	98.9	110.4	2.4	105.6	74.9	

*Note*: Four traps were used for the collection each night in all seven sites. Figures represent total number of *Culicoides* of each species collected during: 3 nights using 4 traps in Bikoki, Bokwai, Ebam and Mbule; 6 nights and 4 traps in Ediki and 2 nights 4 traps in Nlog while figures in parentheses represent the number of *Culicoides* per trap per night (fly/trap/night) for each species in the various sites

*Abbreviations*: *n*, number of night-traps

### Species of *Culicoides* from larval developmental medium

Fisrt and second stage decaying banana/plantain stems (five stems per site) constituted the larval developmental medium. Upon isolation of larvae and rearing, three species, *C. milnei*, *C. grahamii* and *C. inornatipennis*, were common to all the sampled study sites (Table 3). The highest percentage emergence was achieved from larval developmental medium collected from Mbule and the lowest from Bokwai. In general, *C. inornatipennis* was the first (6 days) to emerge and was the most abundant (46.5%; *n* = 1307), while *C. milnei* was the last (10 days) to emerge and the least abundant (11.5%; *n* = 303). Emerging *Culicoides* species were monitored over a 14-day period.

**Table 3:** Percentage emergence of *Culicoides* species larvae isolated from plantain stems in four study sites of the South-West region of Cameroon

Site	No. of larvae isolated	Emerged species	No. of emerged adults	Percentage emergence
Bokwai	1050	*C. milnei*	64	9.5^a^
		*C. grahamii*	198	29.6^a^
		*C. inornatipennis*	408	60.9^a^
Total	1050	–	670	63.8^b^
Mbule	523	*C. milnei*	04	0.8^a^
		*C. grahamii*	298	63.0^a^
		*C. inornatipennis*	171	36.1^a^
Total	523	–	473	90.4^b^
Nlog	1000	*C. grahamii*	204	28.1^a^
		*C. inornatipennis*	523	71.9^a^
Total	1000	–	727	72.7^b^
Ediki	820	*C. milnei*	235	30.6^a^
		*C. grahamii*	327	42.6^a^
		*C. inornatipennis*	205	26.7^a^
Total	820	–	767	93.5^b^
Overall Total	3393	–	2637	77.7

^a^Percentage based on total number of emerged adult flies

^b^Percentage based on the total number of isolated larvae which were then kept in culture

### Anthropophilic *Culicoides* species as determined with the HLC technique

Of the eight species collected in this survey, only four (*C. inornatipennis*, *C. fulvithorax*, *C. grahamii* and *C. milnei*) were collected from humans (Table 4). *Culicoides grahamii* was the most anthropophylic species with a morning fly/person/hour ratio of 48.5 and an evening fly/person/hour ratio of 90.5. This was seconded by *C. milnei*. *Culicoides inornatipennis* and *C. fulvithorax* had the lowest fly person ratios in the evening.

**Table 4:** *Culicoides* species collected during morning and evening periods in 4 sites using the HLC technique

Species	Site								Total collected in the morning	Total collected in the evening	Total collection (%)	C/m/h (morning)	C/m/h (evening)
	Bokwai		Ediki		Mbule		Nlog						
	Morning	Evening	Morning	Evening	Morning	Evening	Morning	Evening					
*C. inornatipennis*	2	27	12	4	64	0	0	0	78	31	109 (1.6)	1.6	0.6
*C. fulvithorax*	0	0	5	8	0	0	0	0	5	8	13 (0.2)	0.1	0.2
*C. grahamii*	896	3147	5	2	1420	967	9	230	2330	4346	6676 (96.5)	48.5	90.5
*C. milnei*	3	0	44	64	10	0	0	0	57	64	121 (1.7)	1.2	1.33
Total	901	3174	66	78	1494	967	9	230	2470	4449	6919 (100)	51.5	92.7

*Notes*: Number of collectors: 4; Number of working hours/morning: 3; Number of working hours/evening: 3; Number of collection days: 4; Total number of working hours morning: 12; Total number of working hours evening: 12

*Abbreviation*: C/m/h: number of *Culicoides* per man per hour

In total, 6919 *Culicoides* were collected by the HLC technique with a significantly higher number collected in the evening period (4–7 pm) compared to the morning period (6–9 am) (*χ*^2^ = 69.9, *df* = 4, *P* < 0.0001) at all of the study sites except in Mbule. The number of *Culicoides* per person per hour was higher in the evening (92.7) when compared to the morning (51.5) periods (Table 4).

### Susceptibility of *C. milnei* to *M. perstans*

**Fig. 3:**
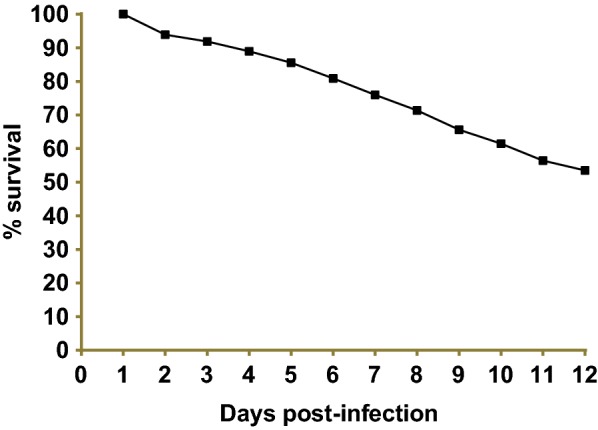
Engorged *Culicoides* survival curve following rearing in the laboratory (note a steady reduction of survival rate over time)

Engorged *Culicoides* (*n* = 2100) were collected with a drop trap and maintained in the laboratory for 12 days. *Culicoides milnei* was the most abundant species collected, followed by *C. grahamii*, *C. inornatipennis*, *C. fulvithorax* and *C. neavei* (Table 5). During the laboratory maintenance period, survival of the midges decreased gradually from the 3rd day post-infection (Fig. 3, Additional file 1: Table S1). After 12 days of maintenance, 53.4% (*n* = 1091) of engorged flies had survived and were dissected to monitor for the presence of *M. perstans* infective larvae. Upon dissection, *C. milnei* was the main species that allowed the development of ingested microfilariae of *M. perstans* to third-stage infective larvae. Out of 807 *C. milnei* dissected, 333 were infected and 584 infective larvae were recovered giving an output of 1.75 L3s/infected fly. Only 7 infective larvae were obtained from 5 *C. grahamii* females out of 194 collected from the same donor. In contrast, the *C. inornatipennis*, *C. fulvithorax* and *C. neavei* engorged on the same donor were all negative for infective larvae of *M. perstans* at dissection (Table 5).

**Table 5:** Developmental stages of *Mansonella perstans* recovered from *Culicoides* fed on a microfilaraemic donor and reared in the laboratory for 12 days following infection

Species	No. collected	No. dissected	No. positive (prevalence)	Larval output										
				Total no. of L3 recovered	Head			Thorax			Abdomen			L3/fly
					L1	L2	L3	L1	L2	L3	L1	L2	L3	
*C. inornatipennis*	108	56	0	0	0	0	0	0	0	0	0	0	0	0
*C. fulvithorax*	4	2	0	0	0	0	0	0	0	0	0	0	0	0
*C. grahamii*	373	194	5 (1.3^a^, 2.6^b^)	7	0	0	4	0	0	3	0	0	0	0.04
*C. milnei*	1553	807	333 (21.4^a^, 41.3^b^)	584	0	0	440	0	8	60	0	14	84	1.75
*C. neavei*	62	32	0	0	0	0	0	0	0	0	0	0	0	0
Total	2100	1091 (51.9)	338 (16.1^a^, 31.0^b^)	591	0	7	444	0	8	63	0	14	84	0.54

^a^Prevalence of infection among collected engorged flies

^b^Prevalence of infection among dissected engorged flies

### Hourly *Culicoides* collection using UV-light traps

**Fig. 4:**
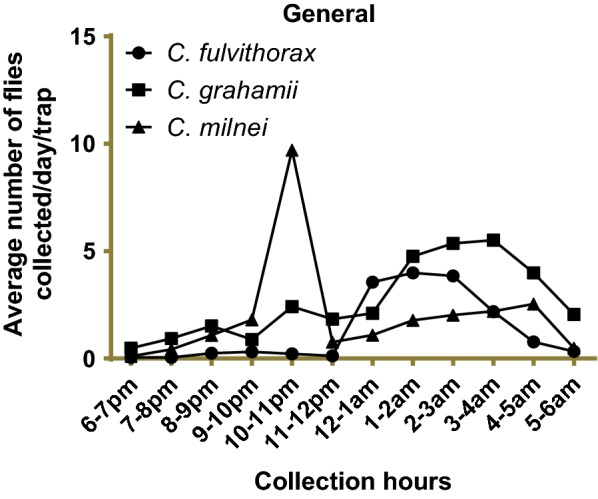
Average number of the most abundant *Culicoides* visiting cycles in all collection sites after 18 nights of collection using four traps

Overall, *C. grahamii* was the most abundant species (average of 200 midges/h) compared to the other 7 captured species (Fig. 4, Additional file 1: Table S2). A peak in the total numbers of *Culicoides* collected was observed between 10 and 11 pm. This peak followed a lower peak observed between 2 and 3 am. *Culicoides fulvithorax* seemed to become active during the second half of the night, peaking between 1 and 3 am. *Culicoides milnei* was characterized by two biting peaks, one in the late evening hours, 10–11 pm, and in the early morning hours between 2 and 4 am (Fig. 4, Additional file 1: Table S2).

**Fig. 5:**
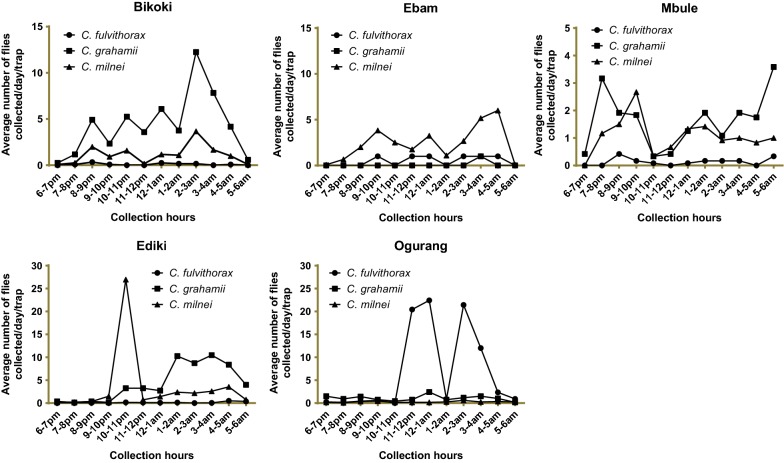
Average number of *Culicoides* species using four light traps during three nights at Bikoki, Ebam, Mbule, Ogurang and six nights at Ediki

Comparing the light trap visiting cycles of the different species of *Culicoides* at the different collection sites (Fig. 5, Additional file 1: Tables S3–S7), *C. grahamii* displayed 2 major biting peaks: one in the early hours of the morning (5–6 am) and the other in the early hours of the evening (7–8 pm) in Mbule (Additional file 1: Table S5). In Ediki, a steady increase in biting intensity was observed from the late hours of the evening towards the early hours of the morning (Additional file 1: Table S6). With the exception of Ebam, *C milnei* was the second most abundant species collected at all sites (Fig. 5, Additional file 1: Tables S3–S7). Two very conspicuous biting peaks were observed in Ebam at 8–10 pm and 4–6 am and relatively very few other *Culicoides* species were collected (Fig. 5, Additional file 1: Table S4). Using the Chi-square test, the hourly trap visiting cycle differed significantly for the different species collected (*χ*^2^ = 3342.3, *df* = 84, *P* < 0.001).

## Discussion

In a defined area, the abundance and diversity of *Culicoides* species strongly depends on the availability and type of breeding sites [8]. Overnight UV-light trap collections indicated that eight *Culicoides* species were common in the surveyed rain forest villages in the South-West region of Cameroon. All eight species had previously been reported in Cameroon [8, 20]. The high number of decaying plantain stems around homes in the seven surveyed communities is no doubt a contributing factor for the abundance of the *Culicoides* species that breed in rotting vegetation. *Culicoides grahamii* was the most abundant species caught overnight and was closely followed by *C. milnei*.

Some authors have suggested that *C. milnei* is identical to *C. austeni* [15, 21]. Ecologically, *C. austeni* is a species native to mangrove and brackish water areas while *C. milnei* is a forest species, as observed in our studies performed here. In 1961, Murphy [22] confirmed that *C. milnei* is a separate species from *C. austeni*. Our observations of the palps, inter-ocular space, the wing patterns and the male genitalia of this species correspond to the description given by Murphy for *C. milnei* [22].

It is well documented that most *Culicoides* species have a crepuscular periodicity, peaking at dusk and dawn [7, 23]. Moreover, crepuscular periodicity can be influenced by nocturnal temperatures and it is suggested that seasonal shifts in the periodicity can occur [24–26]. In this study, *C. grahamii* was dominant in morning, evening and overnight collections whilst *C. milnei* was mainly collected during overnight collections (Additional file 1: Table S8). These findings confirm those of Hopkins [15] that indicated *C. milnei* is essentially a nocturnal species. However, our results differ with regard to *C. grahamii* since they found it to be only a diurnal species but here, this species surpassed all others in all the collection techniques used both during the day and night, except with the drop trap where *C. milnei*-dominated. Our studies concur with the observations of Vattier-Bernard and colleagues [16] that *C. grahamii* has a high biting rate in the early morning and evening hours although in that study there were no overnight collections.

To determine the breeding sites of the different species we investigated first and second stage decaying plantain and banana stems. Three *Culicoides* species, *C. milnei*, *C. grahamii* and *C. inornatipennis*, were common to almost all the sites with *C. inornatipennis* being the most abundant species, while the least was *C. milnei*. All these species were collected with the UV-light traps. Hopkins [15] also reported *C. milnei* and *C. grahamii* in decaying banana and plantain stems and also observed *C. inornatipennis* in third stage vegetation. In this study, the majority of breeding material was collected in the first and second stages of decay. Although we did not separate the material according to the stage of decay, the second stage was relatively more productive compared to the first. We had more of *C. grahamii* than *C. milnei* from this pool of material. Previous studies have shown that *C. milnei* prefers to breed in first rather than second stage decaying material and moreover, it is rare to find this species in third stage material [13]. Likewise, *C. grahamii* also prefers to breed in first stage material [15]. *Culicoides inornatipennis* on the other hand breeds in rot holes in addition to third stage decaying banana and plantain stems which is in contrast to most non-anthropophilic species which prefer moist soil microhabitats of various kinds, including rubbish heaps and dung [15, 27, 28]. An advantage at our study sites lies in the year-round planting and harvesting of bananas and plantains which provides continual breeding sites for *Culicoides* and, in turn, has an implication on yearly fly abundance. Only four anthropophilic species, *C. inornatipennis*, *C. fulvithorax*, *C. grahamii* and *C. milnei*, were collected in the early morning and evening hours. Species diversity was greater using the miniature UV-light trap compared to HLC (Additional file 1: Table S8). When determining the importance of a particular vector species and related dynamics of disease transmission, HLC appears to be a more suitable approach. Most vector species are polyphagous and some midges attracted to light may not normally bite humans and therefore will have no role in disease transmission. Agbolade et al. [29] used HLC methods to distinguish *C. fulvithorax* as the vector of *M. perstans* in the northern area of Western Nigeria. White [30] also captured *C. fulvithorax*, *C. grahamii* and *C. milnei* as anthropophilic species. The high diversity of *Culicoides* species captured with UV-light traps indicates that these serve as a great attractant to midges. It will, however, be difficult to determine whether these species are disease vectors without further investigation [31].

Vector competence refers to the ability of arthropods to acquire, maintain, and transmit pathogenic agents [32]. For example, besides the transmission of filarial parasites, studies in Tunisia, South Africa, Senegal and Nigeria reported that *Culicoides* spp. are able to transmit viruses [33–36]. However, to our knowledge, until now there have been no reports of transmission of viruses from *Culicoides* spp. in Cameroon. In regard to filariae, upon dissection of the engorged specimens, *C. milnei* was the most competent at permitting the development of the infective larval stages from the ingested microfilariae. From 807 fed *C. milnei*, 333 (41.3%) flies were found to harbor *M. perstans* infective larvae, compared to 5 (2.6%) out of 194 fed *C. grahamii.* In 1952, Hopkins [15] did not find any developing larvae of *M. perstans* in 1500 engorged *C. grahamii*. Hopkins & Nicholas [13] also engorged 418 *C. grahamii* on an *M. perstans* donor but found only two flies with infective stage larvae upon dissection. The current data supports those earlier findings since we found only a few infective larvae in *C. grahamii* that had fed on the *M. perstans* donors. Therefore, although the larvae are yet to be confirmed molecularly, *C. grahamii* seems to be an inefficient vector of *M. perstans* infections. Our study therefore confirms the works of Sharp [9, 10] and Hopkins & Nicholas [13] who first made an association between *A. perstans* and the possible vectors. The results of the present study confirm that *C. grahamii* is a poorly competent vector of *M. perstans* while *C. milnei* is a highly competent vector of *M. perstans* [37]. This confirmation on the potential role of *C. milnei* in the development of *M. perstans*, six decades after the first observation by Hopkins & Nicholas [13], has several implications, with the first being the possibility of carrying out surveys on the transmission of *M. perstans* targeting *C. milnei* as the major vector. However, before such programmes can begin, it is important to confirm the observations made here in South-West Cameroon with other bioecological zones of Cameroon. One impediment for the study of transmission of *M. perstans* remains the tedious microscopic dissection of *Culicoides*. Future studies including PCR identification/confirmation of the *M. perstans* vector would constitute a major step for the transmission survey of mansonellosis. Another important finding of this study is that the major potential vector of *M. perstans*, *C. milnei*, is a nocturnal species. It is known from other filariae that parasitize humans that the abundance of blood-dwelling microfilariae correlates with whether the vector bites during the day (*Loa loa*, [38]) or night (*Wuchereria bancrofti*, [39]). *Mansonella perstans* is considered a diurnal species meaning that microfilariae are potentially circulating during day and night [40, 41]. The biting behavior of *C. milnei*, as observed in this study, contradicts the possible diurnal periodicity of *M. perstans* microfilariae since they appeared more abundantly in the peripheral blood during the day. Indeed, in a recent survey for lymphatic filariasis carried out in 31 health districts in the forested area of Cameroon, where we collected blood from the same individuals during the day and night, we observed that the microfilarial load of *M. perstans* was systematically higher during night than during day collection (unpublished data). This apparently contradictory information calls for further studies to clarify the periodicity of microfilariae of *M. perstans* in the peripheral blood in humans and correlate it with the biting behaviors of the *Culicoides* vectors. Such clarification would be necessary to know which is the most convenient period (day or night) for new epidemiological surveys of mansonellosis.

## Conclusions

This study has confirmed observations made 60 years ago on the biodiversity, biting behavior and breeding habitats of *Culicoides* species in South-West Cameroon. It has clarified the important role which *C. milnei* play in the transmission of *M. perstans*, giving the opportunity to target this species for studies on the transmission of mansonellosis in Cameroon and elsewhere.

## Additional file

**Additional file 1: Table S1.** Survival of *Culicoides* species engorged in human volunteer in the laboratory rearing condition over a 12-day period (see Fig. 3). **Table S2.** Overall UV-light trap visiting cycle of *Culicoides* spp. of the South-West region (see Fig. 4)**. Table S3.** UV-light trap visiting cycle of *Culicoides* spp. in Bikoki collection point in the South-West region of Cameroon (see Fig. 5). **Table S4.** UV-light trap visiting cycle of *Culicoides* spp. in Ebam collection point in the South-West region of Cameroon (see Fig. 5). **Table S5.** UV-light trap visiting cycle of *Culicoides* spp. in Mbule collection point in the South-West region of Cameroon (see Fig. 5). **Table S6.** UV-light trap visiting cycle of *Culicoides* spp. in Ediki collection point in the South-West region of Cameroon (see Fig. 5). **Table S7.** UV-light trap visiting cycle of *Culicoides* spp. in Ogurang collection point in the South-West region of Cameroon (see Fig. 5). **Table S8.** Human landing catches of *Culicoides* species in Ediki collection point in the South-West region of Cameroon.

## Abbreviations

HLC: human landing catch
MDA: mass drug administration
UV: ultraviolet
