# Supplementary material for: Update on the biology and ecology of Culicoides species in the South-West region of Cameroon with implications on the transmission of Mansonella perstans
Source: Parasit Vectors. 2019 Apr 11;12:166. doi: 10.1186/s13071-019-3432-9 (PMC6460808; doi:10.1186/s13071-019-3432-9)
Supplement: Supplementary file 1 — Additional file 1: Table S1. Survival of Culicoides species engorged in human volunteer in the laboratory rearing condition over a 12-day period (see Fig. 3). Table S2. Overall UV-light trap visiting cycle of Culicoides spp. of the South-West region (see Fig. 4). Table S3. UV-light trap visiting cycle of Culicoides spp. in Bikoki collection point in the South-West region of Cameroon (see Fig. 5). Table S4. UV-light trap visiting cycle of Culicoides spp. in Ebam collection point in the South-West region of Cameroon (see Fig. 5). Table S5. UV-light trap visiting cycle of Culicoides spp. in Mbule collection point in the South-West region of Cameroon (see Fig. 5). Table S6. UV-light trap visiting cycle of Culicoides spp. in Ediki collection point in the South-West region of Cameroon (see Fig. 5). Table S7. UV-light trap visiting cycle of Culicoides spp. in Ogurang collection point in the South-West region of Cameroon (see Fig. 5). Table S8. Human landing catches of Culicoides species in Ediki collection point in the South-West region of Cameroon. [file 13071_2019_3432_MOESM1_ESM.docx]

**Additional file 1**

**Table S1.** Survival of *Culicoides* spp engorged in human volunteer in the laboratory rearing condition over a 12 days period (see Fig. 3)

| **Days** | **Alive (*n*)** | **Survival (%)** | **Dead (N)** | **Mortality (%)** |
| --- | --- | --- | --- | --- |
| 1 | 1806 | 100 | 0 | 0 |
| 2 | 1696 | 93.91 | 110 | 6.09 |
| 3 | 1659 | 91.86 | 147 | 8.14 |
| 4 | 1606 | 88.93 | 200 | 11.07 |
| 5 | 1545 | 85.55 | 261 | 14.45 |
| 6 | 1461 | 80.9 | 345 | 19.1 |
| 7 | 1372 | 75.97 | 434 | 24.03 |
| 8 | 1289 | 71.37 | 517 | 28.63 |
| 9 | 1185 | 65.61 | 621 | 34.39 |
| 10 | 1110 | 61.46 | 696 | 38.54 |
| 11 | 1019 | 56.42 | 787 | 43.58 |
| 12 | 966 | 53.49 | 840 | 46.51 |

Table S2 Overall UV-light trap visiting cycle of *Culicoides* spp of the South-West region (see Fig. 4)

|  | Species | | | | | | | | Total |
| --- | --- | --- | --- | --- | --- | --- | --- | --- | --- |
| Collection hours | *C. bedfordi* | *C. inornatipennis* | *C. fulvithorax* | *C. grahamii* | *C. imicola* | *C. kumbaensis* | *C. milnei* | *C. neavei* |  |
|  | 0 | 315 | 25 | 576 | 1 | 0 | 388 | 1 | 1306 |
| 6-7pm | 2 | 7 | 4 | 35 | 0 | 2 | 8 | 4 | 62 |
| 7-8pm | 5 | 9 | 4 | 67 | 0 | 23 | 31 | 7 | 146 |
| 8-9pm | 8 | 30 | 18 | 109 | 0 | 20 | 78 | 17 | 280 |
| 9-10pm | 7 | 14 | 23 | 64 | 0 | 18 | 130 | 9 | 265 |
| 10-11pm | 2 | 30 | 16 | 174 | 0 | 14 | 699 | 17 | 952 |
| 11-12pm | 1 | 7 | 9 | 132 | 0 | 5 | 55 | 7 | 216 |
| 12-1am | 2 | 15 | 256 | 152 | 0 | 0 | 79 | 8 | 512 |
| 1-2am | 3 | 17 | 286 | 338 | 0 | 3 | 127 | 11 | 785 |
| 2-3am | 4 | 5 | 277 | 386 | 0 | 4 | 146 | 9 | 831 |
| 3-4am | 5 | 12 | 157 | 397 | 0 | 10 | 159 | 14 | 754 |
| 4-5am | 0 | 11 | 56 | 287 | 0 | 2 | 183 | 6 | 545 |
| 5-6am | 2 | 14 | 24 | 148 | 0 | 3 | 35 | 9 | 235 |
| Total | 41 | 486 | 1155 | 2865 | 1 | 104 | 2118 | 119 | 6889 |

**Table S3** UV-light trap visiting cycle of *Culicoides* spp in Bikoki collection point in the South-West region of Cameroon (see Fig. 5)

|  | Species | | | | | | | | Total |
| --- | --- | --- | --- | --- | --- | --- | --- | --- | --- |
| Collection hours | *C. bedfordi* | *C. inornatipennis* | *C. fulvithorax* | *C. grahamii* | *C. imicola* | *C. kumbaensis* | *C. milnei* | *C. neavei* |  |
| 6-7pm | 2 | 1 | 0 | 3 |  | 0 | 1 | 0 | 7 |
| 7-8pm | 2 | 1 | 1 | 14 |  | 0 | 3 | 2 | 23 |
| 8-9pm | 4 | 2 | 4 | 59 |  | 0 | 24 | 4 | 97 |
| 9-10pm | 6 | 3 | 1 | 28 |  | 1 | 11 | 2 | 52 |
| 10-11pm | 2 | 1 | 0 | 63 |  | 0 | 19 | 4 | 89 |
| 11-12pm | 0 | 0 | 0 | 43 |  | 0 | 2 | 0 | 45 |
| 12-1am | 0 | 1 | 3 | 73 |  | 0 | 14 | 1 | 92 |
| 1-2am | 1 | 0 | 2 | 49 |  | 0 | 13 | 0 | 65 |
| 2-3am | 2 | 1 | 2 | 147 |  | 0 | 44 | 0 | 196 |
| 3-4am | 2 | 2 | 0 | 94 |  | 0 | 20 | 1 | 119 |
| 4-5am | 0 | 0 | 1 | 50 |  | 0 | 12 | 0 | 63 |
| 5-6am | 1 | 0 | 0 | 7 |  | 0 | 1 | 0 | 9 |
| Total | 22 | 12 | 14 | 630 |  | 1 | 164 | 14 | 857 |

**Table S4** UV-light trap visiting cycle of *Culicoides* spp in Ebam collection point in the South-West region of Cameroon (see Fig. 5)

|  | Species | | | | | | | | Total |  |
| --- | --- | --- | --- | --- | --- | --- | --- | --- | --- | --- |
| Collection hours | *C. bedfordi* | *C. inornatipennis* | *C. fulvithorax* | *C. grahamii* | *C. imicola* | *C. kumbaensis* | *C. milnei* | *C. neavei* |  |  |
| 6-7pm | 0 | 0 | 1 | 0 |  | 1 | 1 | 1 | 4 | |
| 7-8pm | 3 | 1 | 1 | 1 |  | 0 | 8 | 2 | 16 | |
| 8-9pm | 1 | 1 | 3 | 1 |  | 0 | 24 | 3 | 33 | |
| 9-10pm | 0 | 0 | 13 | 1 |  | 0 | 46 | 4 | 64 | |
| 10-11pm | 0 | 2 | 0 | 1 |  | 0 | 13 | 4 | 20 | |
| 11-12pm | 1 | 3 | 5 | 2 |  | 0 | 30 | 6 | 47 | |
| 12-1am | 0 | 2 | 6 | 0 |  | 0 | 21 | 2 | 31 | |
| 1-2am | 2 | 3 | 12 | 3 |  | 0 | 40 | 9 | 69 | |
| 2-3am | 1 | 2 | 16 | 3 |  | 0 | 32 | 5 | 59 | |
| 3-4am | 2 | 0 | 10 | 11 |  | 0 | 62 | 7 | 92 | |
| 4-5am | 0 | 0 | 15 | 3 |  | 0 | 72 | 5 | 95 | |
| 5-6am | 0 | 0 | 0 | 0 |  | 0 | 1 | 1 | 2 | |
| Total | 10 | 14 | 82 | 26 |  | 1 | 350 | 49 | 532 | |

**Table S5** UV-light trap visiting cycle of *Culicoides* spp in Mbule collection point in the South-West region of Cameroon (see Fig. 5)

|  | Species | | | | | | | | Total | |
| --- | --- | --- | --- | --- | --- | --- | --- | --- | --- | --- |
| Collection hours | *C. bedfordi* | *C. inornatipennis* | *C. fulvithorax* | *C. grahamii* | *C. imicola* | *C. kumbaensis* | *C. milnei* | *C. neavei* |  |  |
| Overnight |  | 33 | 23 | 343 |  | 0 | 365 | 0 | 764 |  |
| 6-7pm |  | 2 | 0 | 5 |  | 1 | 0 | 0 | 8 |  |
| 7-8pm |  | 0 | 0 | 38 |  | 22 | 14 | 0 | 74 |  |
| 8-9pm |  | 5 | 5 | 23 |  | 19 | 18 | 0 | 70 |  |
| 9-10pm |  | 4 | 2 | 22 |  | 17 | 32 | 0 | 77 |  |
| 10-11pm |  | 7 | 2 | 23 |  | 14 | 17 | 3 | 66 |  |
| 11-12pm |  | 3 | 1 | 4 |  | 5 | 4 | 0 | 17 |  |
| 12-1am |  | 2 | 0 | 5 |  | 0 | 8 | 0 | 15 |  |
| 1-2am |  | 3 | 1 | 15 |  | 2 | 16 | 0 | 37 |  |
| 2-3am |  | 1 | 2 | 13 |  | 3 | 11 | 0 | 30 |  |
| 3-4am |  | 4 | 2 | 23 |  | 9 | 12 | 2 | 52 |  |
| 4-5am |  | 7 | 0 | 21 |  | 2 | 10 | 0 | 40 |  |
| 5-6am |  | 9 | 4 | 43 |  | 3 | 12 | 4 | 75 |  |
| Total |  | 80 | 42 | 578 |  | 97 | 519 | 9 | 1325 |  |

**Table S6** UV-light trap visiting cycle of *Culicoides* spp in Ediki collection point in the South-West region of Cameroon (see Fig. 5)

|  | Species | | | | | | | | Total |
| --- | --- | --- | --- | --- | --- | --- | --- | --- | --- |
| Collection hours | *C. bedfordi* | *C. inornatipennis* | *C. fulvithorax* | *C. grahamii* | *C. imicola* | *C. kumbaensis* | *C. milnei* | *C. neavei* |  |
| 6-7pm |  | 4 | 0 | 9 |  | 0 | 4 |  | 17 |
| 7-8pm |  | 5 | 0 | 3 |  | 1 | 5 |  | 14 |
| 8-9pm |  | 5 | 1 | 9 |  | 1 | 8 |  | 24 |
| 9-10pm |  | 6 | 0 | 4 |  | 0 | 36 |  | 46 |
| 10-11pm |  | 16 | 4 | 78 |  | 0 | 647 |  | 745 |
| 11-12pm |  | 1 | 3 | 78 |  | 0 | 17 |  | 99 |
| 12-1am |  | 6 | 2 | 65 |  | 0 | 34 |  | 107 |
| 1-2am |  | 9 | 3 | 246 |  | 0 | 57 |  | 315 |
| 2-3am |  | 0 | 0 | 209 |  | 0 | 52 |  | 261 |
| 3-4am |  | 5 | 1 | 251 |  | 0 | 62 |  | 319 |
| 4-5am |  | 2 | 12 | 201 |  | 0 | 85 |  | 300 |
| 5-6am |  | 4 | 9 | 96 |  | 0 | 18 |  | 127 |
| Total |  | 63 | 35 | 1249 |  | 2 | 1025 |  | 2374 |

**Table S7** UV-light trap visiting cycle of *Culicoides* spp in Ogurang collection point in the South-West region of Cameroon (see Fig. 5)

|  | Species | | | | | | | | Total | |
| --- | --- | --- | --- | --- | --- | --- | --- | --- | --- | --- |
| Collection hours | *C. bedfordi* | *C. inornatipennis* | *C. fulvithorax* | *C. grahamii* | *C. imicola* | *C. kumbaensis* | *C. milnei* | *C. neavei* |  |  |
| 6-7pm | 0 | 0 | 3 | 18 |  | 0 | 2 | 3 | 26 |  |
| 7-8pm | 0 | 2 | 2 | 11 |  | 0 | 1 | 3 | 19 |  |
| 8-9pm | 3 | 17 | 5 | 17 |  | 0 | 4 | 10 | 56 |  |
| 9-10pm | 1 | 1 | 7 | 9 |  | 0 | 5 | 3 | 26 |  |
| 10-11pm | 0 | 4 | 10 | 9 |  | 0 | 3 | 6 | 32 |  |
| 11-12pm | 0 | 0 | 0 | 5 |  | 0 | 2 | 1 | 8 |  |
| 12-1am | 2 | 4 | 245 | 9 |  | 0 | 2 | 5 | 267 |  |
| 1-2am | 0 | 2 | 269 | 30 |  | 1 | 2 | 3 | 307 |  |
| 2-3am | 1 | 1 | 257 | 14 |  | 1 | 7 | 4 | 285 |  |
| 3-4am | 1 | 1 | 144 | 18 |  | 1 | 3 | 4 | 172 |  |
| 4-5am | 0 | 2 | 28 | 12 |  | 0 | 4 | 1 | 47 |  |
| 5-6am | 1 | 1 | 11 | 2 |  | 0 | 3 | 4 | 22 |  |
| Total | 9 | 35 | 981 | 154 |  | 3 | 38 | 47 | 1267 |  |

**Table S8** Human landing catches visiting cycle of *Culicoides* spp in Ediki collection point in the South-West region of Cameroon

| Collection hours | *C. grahamii* | *C. milnei* | Total |
| --- | --- | --- | --- |
| 6-7pm | 0 | 2 | 2 |
| 7-8pm | 0 | 6 | 6 |
| 8-9pm | 0 | 19 | 19 |
| 9-10pm | 0 | 18 | 18 |
| 10-11pm | 2 | 12 | 14 |
| 11-12pm | 0 | 7 | 7 |
| 12-1am | 0 | 12 | 12 |
| 1-2am | 0 | 7 | 7 |
| 2-3am | 1 | 8 | 9 |
| 3-4am | 0 | 8 | 8 |
| 4-5am | 1 | 2 | 3 |
| 5-6am | 3 | 7 | 10 |
| Total | 7 | 108 | 115 |
